# Supplementary material for: A general strategy for generating expert-guided, simplified views of ontologies
Source: Sci Data. 2026 Jan 9;13:75. doi: 10.1038/s41597-025-06383-w (PMC12827952; doi:10.1038/s41597-025-06383-w)
Supplement: Supplementary file 1 — Figure S1 [file 41597_2025_6383_MOESM1_ESM.pdf]

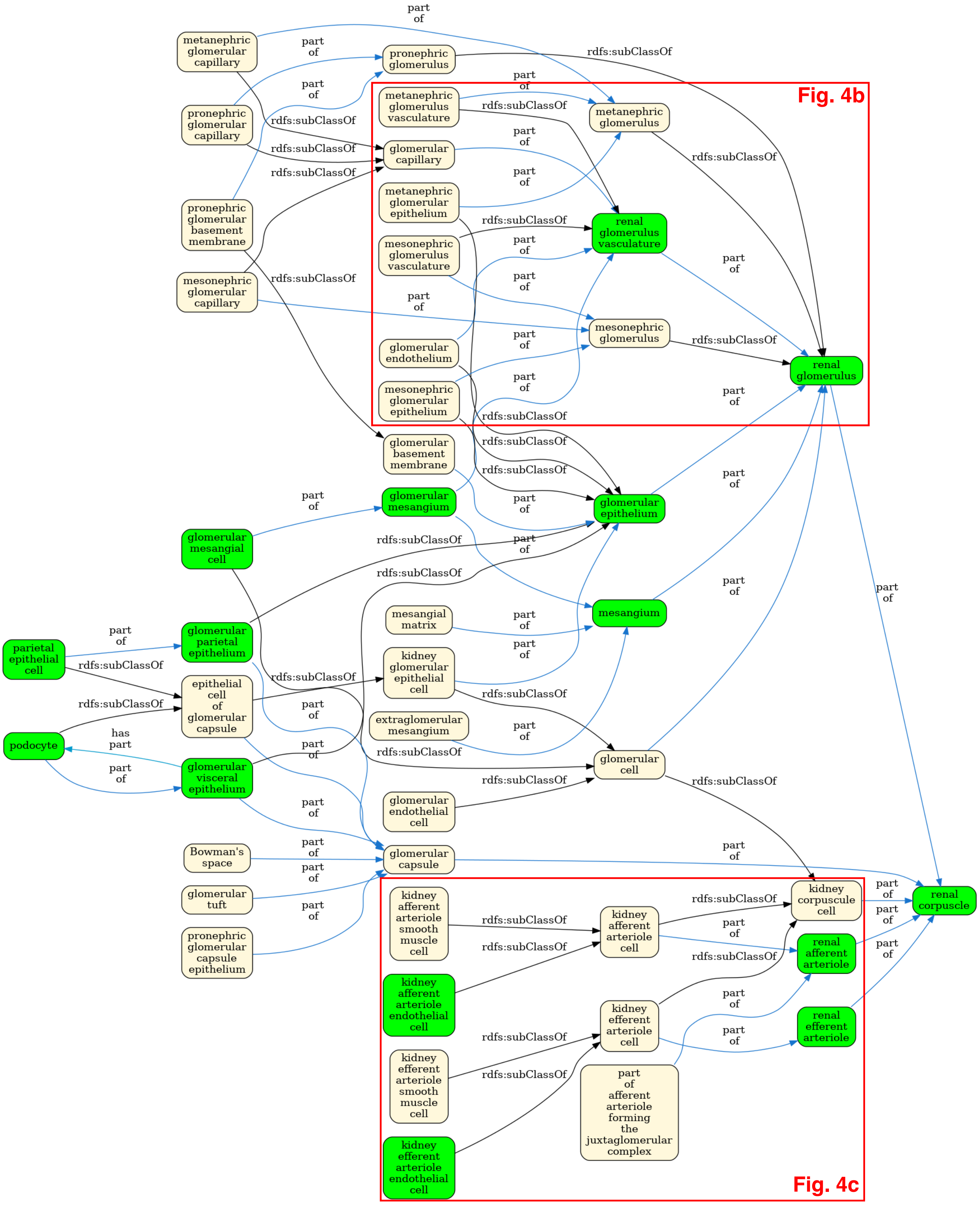

Fig. S1 Uberon/CL ontology graph for the renal corpuscle with terms referenced in the HRA kidney ASCT+B table in green, including the renal corpuscle cell types illustrated in Fig. 4a. This illustrates the complexity of the Uberon graph compared to the needs of the HRA. Enlarged views of selected regions of the graph are provided in Fig. 4b and Fig. 4c.
